# Comparative Study on the Effect of Vitamin D Deficiency on Platelet Indices in COVID‐19 and Hepatitis C Patients; an Observational Comparative Study

**DOI:** 10.1002/hsr2.71945

**Published:** 2026-02-28

**Authors:** Mohammad Motaghi, Kazem Ghaffari, Mahroo Mansori, Ali Ghasemi

**Affiliations:** ^1^ Department of Biochemistry and Hematology Semnan University of Medical Sciences Semnan Iran; ^2^ Department of Hematology and Blood Transfusion Sciences, School of Allied Medical Sciences Tehran University of Medical Sciences Tehran Iran; ^3^ Department of Basic and Laboratory Sciences Khomein University of Medical Sciences Khomein Iran; ^4^ Students Scientific Research Center Tehran University of Medical Sciences Tehran Iran; ^5^ Cancer Research Center Semnan University of Medical Sciences Semnan Iran

**Keywords:** COVID‐19, HCV, MPV, platelet counts, vitamin D

## Abstract

**Background and Aims:**

Despite growing interest in the role of vitamin D in various infectious diseases, limited evidence exists regarding its association with platelet (PLT) parameters in patients with COVID‐19 and Hepatitis C Virus (HCV) infection. This study aims to assess the relationship between serum vitamin D levels and PLT indices in patients with COVID‐19 and chronic HCV.

**Methods:**

The study included 113 patients with confirmed COVID‐19 and 97 patients with chronic HCV infection. All participants were classified into two subgroups based on their vitamin D status (deficient vs. non‐deficient). The chi‐square test (χ2) or Fisher exact test was used to compare the results between the two groups.

**Results:**

Patients with COVID‐19 had significantly lower levels of vitamin D compared with HCV patients (*p* < 0.001). PLT counts were lower in the COVID‐19 group, and MPV, PDW, and inflammatory markers (PLR, MPR, and MLR) were significantly higher. Also, a significant negative correlation was observed between vitamin D levels and MPV in both groups (*p* < 0.001). In patients with vitamin D deficiency, PLT and inflammatory changes were more pronounced in COVID‐19 than in HCV.

**Conclusion:**

The results of this study showed that COVID‐19 patients had lower vitamin D levels, lower PLT counts, and higher indices of platelet activation compared with HCV patients. These changes are associated with the severity of the inflammatory response in COVID‐19 and could be part of the mechanisms that cause thrombotic complications in this disease.

## 1 Introduction

Viral infections are frequently accompanied by hematological and inflammatory disturbances, among which platelet abnormalities play a central role in disease progression and complications. Both acute and chronic viral infections are associated with immune dysregulation and platelet activation, contributing to thrombotic and inflammatory complications [1, 2, 3, 4]. COVID‐19, as an acute viral illness and hepatitis C virus (HCV) infection, as a chronic inflammatory disease are each associated with platelet abnormalities but the mechanisms driving these effects are different [5, 6, 7].

Vitamin D deficiency is reaching epidemic proportions on a global scale and has been increasingly identified as a modulating factor of the immune response, inflammatory processes, and hemostasis. Recent studies have reported that platelets express vitamin D receptors, implying a direct effect on platelet activation and function. Changes in vitamin D levels have also been associated with changes in platelet indices such as mean platelet volume (MPV) and platelet count, the information of which reflect platelet reactivity [2, 8, 9, 10]. Although some studies have investigated the association of vitamin D deficiency with platelet indices in different clinical circumstances, data related to viral infections are lacking. In addition, comparative evidence assessing the impact of vitamin D deficiency on platelet parameters in acute and chronic viral infections is limited. Although COVID‐19 and HCV differ fundamentally in disease duration and pathophysiology—representing acute and chronic infections, respectively—both conditions share common features of immune activation, inflammatory burden, and platelet dysregulation. Given the emerging evidence linking vitamin D status to platelet behavior, a comparative evaluation of platelet indices in the context of vitamin D deficiency across these two distinct viral infections may help delineate shared versus disease‐specific patterns. Such an approach may enhance understanding of the interplay between vitamin D, platelet activation, and viral disease processes, and may provide clinically relevant insights for risk stratification and supportive management strategies.

Therefore, this observational comparative study aimed to investigate the association between serum vitamin D levels and platelet indices in patients with COVID‐19 and chronic HCV infection, and to compare these parameters between individuals with and without vitamin D deficiency in each group. Additionally, we sought to explore the potential clinical relevance of platelet indices in the context of vitamin D deficiency across acute and chronic viral infections.

## 2 Methods and Materials

### 2.1 Research Participants and Biochemical Analyses

The study enrolled 113 patients with confirmed COVID‐19 and 97 patients with chronic HCV infection who were admitted to the hospital. Based on vitamin D status, each disease group was further stratified into subgroups with and without deficiency. Serum vitamin D concentrations were classified as either sufficient or deficient, using the conventional cutoff value of 20 ng/mL [11]. All HCV patients tested negative for COVID‐19, and conversely, all COVID‐19 patients tested negative for HCV. The study population comprised only those individuals who had undergone biochemical assessments as well as a baseline complete blood count (CBC). An automated hematology analyzer (Mindray BC‐6800, China) was used to measure platelet count, platelet distribution width (PDW), MPV, plateletcrit (PCT), and lymphocyte count. Aspartate aminotransferase (AST) was assessed via a spectrophotometric assay (Pars Azmun, Iran), and serum 25(OH) D levels were determined using ELISA (EUROIMMUN, Germany). Derived indices included platelet‐to‐lymphocyte ratio (PLR), monocyte‐to‐platelet ratio (MPR), monocyte‐to‐lymphocyte ratio (MLR), MPV/PCT, PDW/PCT, PDW/PLT, APRI, as well as BMI and interaction terms: vitamin D × MPV, vitamin D × PLT, and vitamin D × PDW.

### 2.2 Inclusion and Exclusion Criteria

Patients with laboratory‐confirmed COVID‐19 (by RT‐PCR) or chronic hepatitis C virus (HCV) infection who were aged ≥ 18 years were eligible for inclusion in this study. Participants were required to have available baseline laboratory data, including serum vitamin D levels and platelet indices, at the time of enrollment. Patients with conditions known to affect platelet count or vitamin D metabolism were excluded, including cardiovascular diseases requiring antiplatelet therapy, pregnancy or pregnancy‐related complications, malignancies, chronic inflammatory or autoimmune disorders, uncontrolled diabetes mellitus, and other chronic liver diseases such as primary sclerosing cholangitis, primary biliary cholangitis, Wilson's disease, and hemochromatosis. Additional exclusion criteria included acute pancreatitis or pancreatic disorders, co‐infection with multiple hepatitis viruses, and the use of antiplatelet agents, nonsteroidal anti‐inflammatory drugs, or vitamin D supplementation prior to enrollment.

### 2.3 Statistical Analysis

Statistical analyses were performed using the Statistical Package for the Social Sciences (SPSS) software, version 25.0 (IBM Corp., Armonk, NY, USA). Continuous variables were expressed as mean ± standard deviation (SD), while categorical variables were presented as counts and percentages. Comparisons between categorical variables were conducted using the Chi‐square (χ²) test. Differences in continuous variables between groups were assessed using one‐way analysis of variance (ANOVA), as appropriate. Pearson's correlation coefficient was applied to evaluate the strength and direction of associations between serum vitamin D levels and platelet indices. A two‐tailed *P* value of less than 0.05 was considered statistically significant.

## 3 Results

The mean age of COVID‐19 patients was 49.2 ± 13.9 years, while those with HCV had a mean age of 44.2 ± 11.7 years. Men accounted for about 60% of the COVID‐19 group and approximately 56% of the HCV group, demonstrating a comparable gender distribution across both cohorts. Serum vitamin D levels were markedly lower in COVID‐19 patients, averaging 23.4 ng/mL ± 7.8, compared to 27.6 ng/mL ± 8.2 in HCV patients, with this difference being highly significant (*p* < 0.001). Thrombocytopenia was present in 13.3% of individuals with COVID‐19% and 11.5% of those with HCV. No notable differences were observed between the groups in terms of gender, body weight, or BMI (*p* > 0.05). However, vitamin D levels varied significantly, with COVID‐19 patients showing lower concentrations (see Table 1).

**Table 1:** Demographic and clinical characteristics of patients.

Characteristics	COVID‐19 patients (*N* = 113)	HCV patients (*N* = 97)	*P*
Gender, M/F	68/45	54/43	0.624
Age, years ± SD	49.2 ± 13.9	44.2 ± 11.7	0.003
Mean weight ± SD (kg)	73.6 ± 28.2	72.1 ± 30.1	0.735
Mean body mass index ± SD (kg/m2)	26.4 ± 5.1	25.1 ± 4.4	0.083
Vit D (ng/mL)	23.4 ± 7.8	27.6 ± 8.2	< 0.001
Vitamin D Deficiency, *n* (%)	75 (66.3)	58 (59.7)	0.340

*Note:* Data are No. (%) unless otherwise indicated.

Abbreviations: SD, standard of deviation; *n*, number.

Patients diagnosed with COVID‐19 exhibited a considerably lower average platelet count compared to individuals with HCV infection (*p* = 0.012). Conversely, their mean platelet volume (MPV) was significantly higher (*p* = 0.008). Indicators of inflammation such as PLR, MPR, and MLR were also markedly increased in the COVID‐19 group, indicating a more pronounced inflammatory response. Additionally, markers associated with platelet activation—including platelet distribution width (PDW), MPV to platelet count ratio (MPV/PCT), and PDW to PCT ratio—were significantly elevated among COVID‐19 patients. While the combined marker Vitamin D multiplied by PDW did not show a significant difference, both Vitamin D multiplied by MPV and Vitamin D multiplied by PLT levels were significantly reduced in COVID‐19 cases. These results underscore notable differences in platelet behavior and inflammatory status between the two patient groups (Table 2).

**Table 2:** Comparison of platelet parameters.

Laboratory values	COVID‐19 patients (*N* = 113)	HCV Patients (*N* = 97)	*p*
Mean PLT ± SD (×10^3^/μl)	185.2 ± 65.4	210.5 ± 58.7	0.012
Min‐Max	90–320	110–340	
MPV ± SD, fL	11.2 ± 1.1	10.6 ± 1.0	0.008
MPR	0.060 ± 0.015	0.050 ± 0.012	0.015
Mean lymphocyte ± SD (×10^3^/μl)	1.1 ± 0.4	1.6 ± 0.5	< 0.001
MLR	0.38 ± 0.12	0.31 ± 0.10	0.021
PLR	168.3 ± 52.7	131.5 ± 48.2	< 0.001
PCT, %	0.19 ± 0.06	0.22 ± 0.07	0.034
MPV/PCT	59.0 ± 12.5	48.2 ± 10.3	0.009
PDW, %	15.8 ± 2.1	13.9 ± 1.8	< 0.001
PDW/PCT	83.2 ± 15.7	63.2 ± 12.4	< 0.001
PDW/PLT	0.085 ± 0.020	0.066 ± 0.018	0.011
Vitamin D* MPV	262.1 ± 45.3	292.6 ± 50.7	0.006
Vitamin D* PLT	4329 ± 1025	5803 ± 1187	< 0.001
Vitamin D* PDW	370.7 ± 62.1	383.6 ± 58.9	0.318
Mean AST ± SD, (IU/L)	42.5 ± 18.7	58.3 ± 22.1	< 0.001
APRI	0.65 ± 0.28	1.12 ± 0.35	< 0.001

Abbreviations: APRI, AST‐to‐PLT ratio index; AST, aspartate aminotransferase; MLR, MPV to lymphocyte ratio; MPR, MPV/platelet count ratio; MPV, mean platelet volume; PCT, plateletcrit; PDW, platelet distribution width; PLR, platelet‐lymphocyte ratio; PLT, platelet count.

* Denote the multiplication operation.

**Figure 1:**
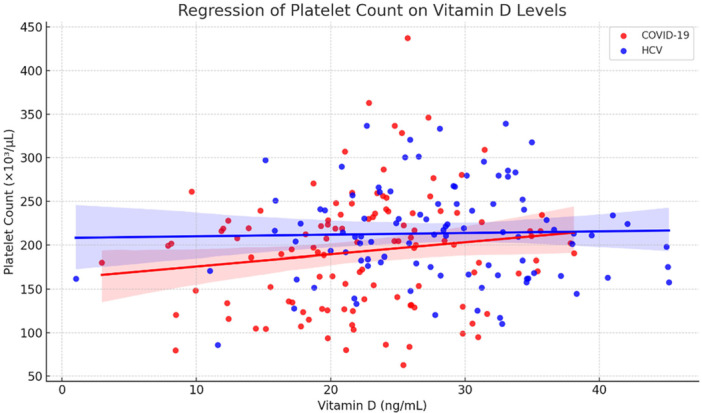
Scatterplot showing the relationship between serum vitamin D levels and platelet count (×10³/μL) in patients with COVID‐19 and HCV. Red and blue regression lines represent linear trends for COVID‐19 and HCV patients, respectively. A weak, non‐significant positive correlation is observed in the COVID‐19 group (*p* = 0.128), while no significant association is evident in the HCV group (*p* = 0.792). *P*‐value was calculated by Pearson's correlation.

**Figure 2:**
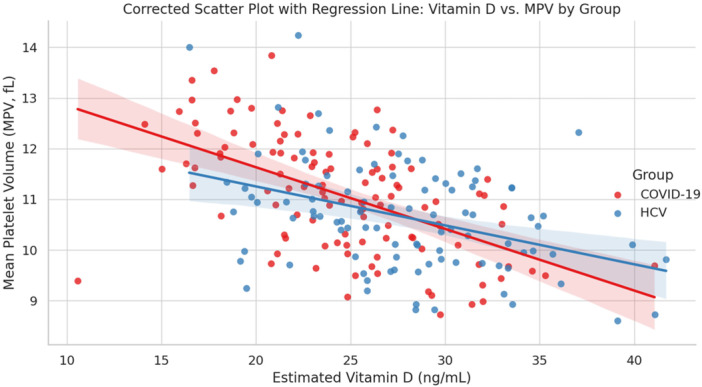
Scatterplot showing the relationship between serum vitamin D levels and MPV in patients with COVID‐19 and HCV. Red and blue regression lines represent linear trends for COVID‐19 and HCV patients, respectively. There is a statistically significant moderate negative correlation between Vitamin D levels and MPV in both patient groups (*p* < 0.001). *P*‐value was calculated by Pearson's correlation.

Pearson's correlation analysis was used to evaluate the relationships between serum vitamin D levels and both platelet count and MPV in patients diagnosed with vitamin D deficiency. As depicted in Figure 1, there was no significant association between vitamin D concentrations and platelet count in either group (*p* = 0.128 and *p* = 0.792, respectively). Conversely, serum vitamin D levels showed a noteworthy inverse correlation with MPV across both groups (*p* < 0.001), as demonstrated in Figure 2.

According to the data summarized in Table 3, COVID‐19 patients who suffered from vitamin D deficiency exhibited a notably lower average platelet count compared to HCV patients also deficient in vitamin D (188.6 ± 54.2 vs. 205.1 ± 52.5; *p* = 0.032). Additionally, the COVID‐19 group showed a significantly increased MPV (11.4 ± 1.0 compared to 10.8 ± 0.9; *p* = 0.020). The PLR was markedly higher in individuals with COVID‐19 (172.6 ± 47.5 vs. 138.9 ± 42.3; *p* < 0.001).

**Table 3:** Comparison of platelet parameters in patients with vitamin D deficiency.

Laboratory values	COVID‐19 Patients with vitamin D deficiency (*N* = 75)	HCV Patients with vitamin D deficiency (*N* = 58)	*p*
Mean PLT ± SD (×10^3^/μl)	188.6 ± 54.2	205.1 ± 52.5	0.032
MPV ± SD, fL	11.4 ± 1.0	10.8 ± 0.9	0.020
MPR	0.064 ± 0.013	0.056 ± 0.011	0.031
Mean lymphocyte ± SD (×10^3^/μl)	1.05 ± 0.35	1.48 ± 0.40	< 0.001
MLR	0.41 ± 0.11	0.33 ± 0.09	0.017
PLR	172.6 ± 47.5	138.9 ± 42.3	< 0.001
PCT, %	0.18 ± 0.05	0.21 ± 0.06	0.039
MPV/PCT	62.4 ± 11.8	51.2 ± 10.1	0.007
PDW, %	16.2 ± 2.0	14.1 ± 1.7	< 0.001
PDW/PCT	87.1 ± 14.3	66.5 ± 11.8	< 0.001
PDW/PLT	0.091 ± 0.018	0.072 ± 0.016	0.010
Vitamin D* MPV	244.5 ± 42.8	280.6 ± 46.9	0.004
Vitamin D* PLT	4065 ± 950	5320 ± 1030	< 0.001
Vitamin D* PDW	353.2 ± 58.5	375.1 ± 55.4	0.109
Mean AST ± SD, (IU/L)	44.8 ± 15.2	59.7 ± 19.8	< 0.001
APRI	0.72 ± 0.25	1.18 ± 0.31	< 0.001

Abbreviations: APRI, AST‐to‐PLT ratio index; AST, aspartate aminotransferase; MLR, MPV to lymphocyte ratio; MPR, MPV/platelet count ratio; MPV, mean platelet volume; PCT, plateletcrit; PDW, platelet distribution width; PLR, platelet‐lymphocyte ratio; PLT, platelet count.

* Denote the multiplication operation.

Moreover, several other platelet‐related and inflammatory markers displayed meaningful differences between the groups: COVID‐19 patients had higher MPR and PDW values (*p* = 0.031 and *p* < 0.001, respectively), while PCT was significantly lower in this group (*p* = 0.039). On the other hand, liver function tests revealed that the HCV group had considerably higher mean AST levels and APRI scores (*p* < 0.001), indicating more extensive liver involvement in these patients.

**Figure 3:**
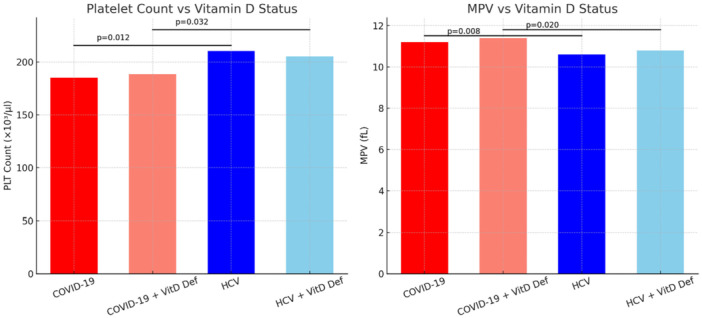
PLT and MPV in COVID‐19 and HCV patients according to vitamin D status. HCV patients showed significantly higher PLT counts compared to COVID‐19, while MPV was significantly higher in COVID‐19 patients (with or without vitamin D deficiency) than in HCV patients.

As shown in Figure 3, patients with HCV infection exhibited notably higher platelet counts compared to individuals diagnosed with COVID‐19 (*p* = 0.012). This distinction persisted regardless of whether patients were vitamin D deficient or not. Conversely, MPV was considerably greater in COVID‐19 patients, including those who also had vitamin D deficiency, when compared to the HCV group (*p* = 0.008 and *p* = 0.020, respectively).

## 4 Discussion

Vitamin D deficiency is a global challenge affecting billions of people. The prevalence of this deficiency is particularly high among Iranian men and women, especially in older age groups [12]. This study was conducted on patients with COVID‐19 and patients with HCV. Serum vitamin D levels in COVID‐19 patients were significantly lower than in HCV patients (23.4 ± 7.8 ng/mL vs. 27.6 ± 8.2 ng/mL, *p* < 0.001). These findings indicate a higher prevalence of vitamin D deficiency in COVID‐19 patients compared to HCV patients.

Analysis of PLT parameters showed that PLT count was significantly lower in COVID‐19 patients than in HCV patients (185.2 ± 65.4 × 10^3^/μL vs. 210.5 ± 58.7 × 10^3^/μL, *p* = 0.012). In addition, MPV was higher in the COVID‐19 group (11.2 ± 1.4 fL vs. 10.6 ± 1 fL, *p* = 0.008). The PLR and the MPR were also significantly higher in COVID‐19 patients (*p* < 0.001 and *p* = 0.015, respectively). These changes suggest a more severe inflammatory state in COVID‐19 patients.

The findings of this study show that patients with COVID‐19 had significantly lower vitamin D levels, a lower PLT count, and higher indices of PLT activation (including MPV, PDW, and their derived ratios) compared to patients with HCV. These results point to a significant role for vitamin D deficiency and PLT changes in the pathophysiology of COVID‐19.

Previous studies have indicated that vitamin D deficiency can be associated with an increased risk of viral infections, including influenza and coronaviruses, with proposed mechanisms involving impaired regulation of innate and adaptive immune responses. Engin et al. reported a link between vitamin D deficiency and acute respiratory infections caused by viruses like influenza and SARS‐CoV‐2, noting that vitamin D plays an immunoprotected role by stimulating antiviral peptides and modulating the inflammatory response [13]. A meta‐analysis also showed that vitamin D supplementation reduces the risk of acute respiratory infections; proposed mechanisms include increased production of antibacterial peptides (such as defensins) and a reduction in pro‐inflammatory cytokines [14].

In the present study, COVID‐19 patients had significantly lower serum vitamin D levels than HCV patients, which is consistent with previous findings [11]. Furthermore, a negative correlation between vitamin D level and MPV suggests that a decrease in vitamin D may be associated with increased PLT activation and changes in blood hemostasis.

In line with our findings, prior evidence suggests that inflammatory and platelet‐related markers play a significant role in the pathophysiology of COVID‐19. A study reported that alterations in inflammatory markers were associated with disease severity, supporting the clinical relevance of surrogate hematological parameters even in the absence of overt thrombotic or bleeding events [15]. Recent evidence suggests that vitamin D supplementation may influence platelet‐related indices and vascular outcomes. For instance, a recent study in patients with type 2 diabetes demonstrated that platelet indices in conjunction with vitamin D levels could predict deterioration of glycemic control and vascular complications, highlighting the potential clinical relevance of vitamin D–mediated platelet modulation [16].

From a hematological standpoint, thrombocytopenia was more common in COVID‐19 patients than in HCV patients. This has been reported in prior studies and is attributed to mechanisms such as direct destruction of platelets by the virus, inhibited bone marrow PLT production, or excessive consumption in inflammatory and thrombotic processes [17, 18]. The increase in MPV and PDW in COVID‐19 patients signifies greater PLT activation, which can elevate the risk of thrombotic events [18]. In contrast, HCV patients more commonly experience chronic liver disorders and secondary PLT reduction due to fibrosis and hypersplenism [19]. Furthermore, inflammatory ratios such as PLR, MPR, and MLR were significantly higher in the COVID‐19 group, indicating a more severe inflammatory response in these patients. This finding aligns with the “cytokine storm” hypothesis in COVID‐19, which is recognized as a primary cause of tissue damage and multi‐organ failure [20]. On the other hand, higher AST levels and APRI scores were observed in HCV patients, indicating greater liver involvement in this group. This finding is consistent with the chronic nature of HCV disease, which leads to persistent inflammation and liver fibrosis [21, 22]. Therefore, it can be concluded that while PLT abnormalities exist in both diseases, they have different underlying mechanisms: in COVID‐19, they are mainly due to acute inflammatory and thrombotic processes, whereas in HCV, they are primarily caused by liver involvement and portal hypertension.

The observed relationship between vitamin D deficiency and PLT changes in COVID‐19 patients suggests that vitamin D may act as a modulator of PLT hemostasis. There is evidence that by inhibiting inflammatory pathways and reducing the production of pro‐inflammatory cytokines, vitamin D can have a protective role against excessive PLT activation [23]. Thus, vitamin D supplementation may be an ancillary strategy in the management of COVID‐19 patients, although more studies are needed to prove its efficacy.

A similar study on 707 COVID‐19 patients found that PLT count and MPV were significantly higher in patients with vitamin D deficiency compared to those without [24]. Similar studies in Korean patients have shown that individuals with vitamin D deficiency typically have lower levels of both PLT and MPV. An inverse correlation was also observed between vitamin D groups and PLT and MPV, such that lower vitamin D levels were associated with lower PLT count and MPV [25]. Another study by Alanli et al. reported an increase in PLT count in individuals with low vitamin D levels [26]. However, the results of various studies on the relationship between MPV and vitamin D are conflicting; some show an inverse relationship, while others find no significant association. For instance, a study on pregnant women with diabetes found that they had low 25‐hydroxyvitamin D3 levels and high MPV, while Cumhur et al. [27] and Alanli et al. [26] found no significant relationship between vitamin D deficiency and MPV in healthy individuals.

One study showed that the vitamin D receptor (VDR) system plays a crucial role in preventing blood clot formation. VDR‐deficient (VDRKO) mice, after lipopolysaccharide injection, showed increased clot formation in various organs regardless of calcium levels. Conversely, vitamin D activation led to increased expression of antithrombotic factors and thrombomodulin genes and decreased expression of thrombogenic factor genes, while VDRKO mice showed the opposite effect [26]. Megakaryocytes, the precursor cells of platelets, possess VDRs, and their activation affects megakaryocyte maturation and proliferation, leading to an increase in PLT count during vitamin D deficiency [28].

Moreover, vitamin D deficiency is associated with an increase in pro‐inflammatory cytokines like IL‐6 and TNF‐α, which can contribute to an increase in MPV [24]. These cytokines may influence hematopoietic stem cells by stimulating megakaryopoiesis and PLT production [29]. Other studies have also shown increased IL‐6 and TNF‐α in COVID‐19 patients [30], suggesting that the cytokine storm may be a reason for increased MPV and PLT in these patients with vitamin D deficiency.

Although some studies have identified PLT as a prognostic marker in sepsis, acute illnesses, and COVID‐19, the results have been varied and contradictory. This could be due to differences in measurement methods, types of anticoagulants, and sample storage times [31]. Differences in patients' clinical conditions, including vitamin D deficiency, gender, and PLT antigen polymorphisms, may also contribute to these inconsistencies.

The association between vitamin D deficiency and unfavorable platelet indices observed in this study may have relevant clinical implications. Given the role of platelet activation in thrombo‐inflammatory pathways, particularly in COVID‐19 and chronic liver disease, assessment of vitamin D status may help identify patients at increased risk of hematological and inflammatory complications. Routine screening for vitamin D deficiency in high‐risk populations, including patients with COVID‐19 and chronic HCV infection, could be considered. While vitamin D supplementation should follow established clinical guidelines, its potential role as an adjunctive supportive measure warrants further investigation in well‐designed interventional studies.

Gender and demographic characteristics may influence vitamin D metabolism, platelet function, and downstream clinical outcomes. Previous studies have shown that sex hormones, particularly estrogen and testosterone, can modulate platelet activation and aggregation, while also affecting vitamin D bioavailability through differences in adipose tissue distribution and hepatic metabolism. Moreover, aging has been associated with reduced cutaneous synthesis of vitamin D and altered platelet reactivity, potentially increasing thrombo‐inflammatory risk. Although gender distribution was comparable between groups in the present study, these biological differences may partially contribute to interindividual variability and should be considered in future studies employing sex‐specific and age‐adjusted analyses [32, 33].

This study has several limitations. First, its cross‐sectional design restricts the ability to determine a causal relationship. Second, the relatively limited sample size may reduce the generalizability of the results. Third, potential confounding variables such as nutritional status, supplement use, and antiviral medications were not fully controlled. Furthermore, only laboratory indices were used, and more detailed clinical assessments, including thrombotic outcomes or disease severity, were not included. Lifestyle‐related factors and detailed nutritional status, which may influence vitamin D levels and platelet parameters, were not directly assessed and should be considered when interpreting the findings. Another limitation of this study is the absence of clinical thrombotic or bleeding events among the study population, which precluded the evaluation of the association between vitamin D levels, platelet indices, and overt clinical outcomes. In addition, the single‐center setting and moderate sample size may restrict the generalizability of the findings.

## 5 Conclusion

In summary, the results of this study showed that COVID‐19 patients, compared with HCV patients with lower vitamin D levels, had reduced PLT counts and higher indices of PLT activation. These alterations appear to be associated with the intensity of the inflammatory response in COVID‐19 and may contribute to the mechanisms underlying thrombotic complications in this disease. Therefore, assessment and correction of vitamin D deficiency may play a role in improving the prognosis of COVID‐19 patients. Further prospective studies with larger sample sizes and interventional designs are recommended.

## Author Contributions

**Mohammad Motaghi:** supervision, conceptualization, writing‐review and editing, writing – original draft, data curation. **Kazem Ghaffari:** conceptualization, supervision, writing‐review and editing, writing – original draft, data curation. **Mahroo Mansori:** conceptualization, writing – original draft, formal analysis, visualization. **Ali Ghasemi:** conceptualization, supervision, writing – original draft, writing – review and editing.

## Ethics Statement

Ethical principles according to the protocol sanctioned by the Ethics Committee at Semnan University of Medical Sciences, Semnan, Iran (IR.SEMUMS.REC.1403.023) and the Declaration of Helsinki. Consent was obtained by first explaining the research's goals to the participants and then asking for their consent.

## Conflicts of Interest

The authors declare no conflicts of interest.

## Transparency Statement

The lead author Ali Ghasemi affirms that this manuscript is an honest, accurate, and transparent account of the study being reported; that no important aspects of the study have been omitted; and that any discrepancies from the study as planned (and, if relevant, registered) have been explained.

## Data Availability

The data regarding this manuscript is made available upon reasonable request from the corresponding author.
